# Predictors of Survival in Patients Aged ≥70 with Glioblastoma: A Time-Dependent Multivariable Analysis

**DOI:** 10.3390/cancers18010178

**Published:** 2026-01-05

**Authors:** Ahmad M. S. Ali, Viraj Parmar, Cathal J. Hannan, Jibril Osman Farah

**Affiliations:** 1 Department of Neurosurgery, The Walton Centre NHS Foundation Trust, Liverpool L9 7LJ, UKcathal.hannan@nhs.net (C.J.H.); jibril.farah1@nhs.net (J.O.F.); 2 Institute of Systems, Molecular, and Integrative Biology, University of Liverpool, Liverpool L69 7BE, UK

**Keywords:** glioblastoma, older patients, surgical resection, adjuvant chemoradiotherapy

## Abstract

**Background:** Glioblastoma (GB) carries a dismal prognosis, with survival outcomes particularly poor in older patients. With the fastest-growing global demographic being those aged over 65, the incidence of GB is expected to rise. **Objective:** To evaluate predictors of survival in patients aged ≥70 years with histologically confirmed GB, focusing on surgical resection, adjuvant therapy, and comorbidities. **Methods:** A retrospective review was performed of all patients aged ≥70 undergoing index surgery for GB between January 2021 and March 2025 at a single tertiary neurosurgical centre. Demographics, pre-operative fitness scores (Karnofsky Performance Status [KPS]., Charlson Comorbidity Index [CCI].), tumour characteristics, extent of resection, adjuvant treatment, and survival were analysed. Tumour volume was estimated using the ABC/2 method. Survival outcomes were assessed using Kaplan–Meier curves and multivariable Cox proportional hazards regression. **Results:** A total of 124 patients aged ≥70 years (median 74 years, range 70–86) were included. Median overall survival was 8 months (IQR 4–15). On multivariable analysis, adjuvant chemoradiotherapy (HR = 0.30, 95% CI 0.17–0.52; *p* < 0.001) and gross total resection (GTR) (HR = 0.41, 95% CI 0.20–0.86; *p* = 0.019) were independently associated with improved survival. Smoking history was associated with increased hazard of death (HR = 2.02, 95% CI 1.07–3.81; *p* = 0.029), an effect robust to multiple sensitivity analyses. No significant associations were found for age, pre-operative KPS, comorbidity index, tumour volume, or methylation status (all *p* > 0.10). Tests for non-proportional hazards indicated that the survival benefit of adjuvant therapy diminished over time (interaction *p* = 0.0002), with early post-operative benefit (HR ≈ 0.35 at 1 month) that attenuated towards unity by 6–12 months. The effects of GTR and smoking were time-invariant. RMST analysis suggested a modest, non-significant absolute survival advantage of GTR over STR (mean difference = 2.0 months at 18 months; *p* = 0.11). After exclusion of early post-operative deaths (<6 weeks), adjuvant therapy (HR = 0.34; *p* < 0.001) and GTR (HR = 0.33; *p* = 0.005) remained independent predictors of improved survival. **Conclusions:** Among patients aged ≥70 years with glioblastoma, adjuvant therapy and extent of resection remain key independent predictors of survival, while smoking is associated with poorer outcomes. The survival benefit of adjuvant chemoradiotherapy is strongest in the early post-operative period and diminishes over time, underscoring the importance of early multidisciplinary intervention. These findings highlight that aggressive multimodal treatment may confer survival advantage even in older patients.

## Simple Summary

Glioblastoma is an aggressive brain tumour with a very poor outlook, particularly in older patients, and its incidence is expected to rise as the population ages. This study reviewed the outcomes of 124 patients aged 70 years or older who underwent surgery for glioblastoma at a single specialist neurosurgical centre between 2021 and 2025, with the aim of identifying factors linked to survival. Overall survival remained limited, with a median survival of around 8 months. Two factors were clearly associated with longer survival. Patients who received chemotherapy and radiotherapy after surgery lived longer than those who did not, with the benefit being strongest in the early months following surgery and gradually reducing over time. In addition, patients in whom all visible tumour could be removed during surgery tended to live longer than those who had only partial tumour removal. In contrast, age within this older group, general fitness before surgery, the presence of other medical conditions, tumour size, and molecular tumour features did not show a clear link with survival. Notably, a history of smoking was associated with poorer survival, even after accounting for other factors. Taken together, these findings suggest that selected patients aged 70 and over can still benefit from active, combined treatment approaches, and that early, coordinated decision-making around surgery and post-operative therapy is important to maximise potential survival benefits in this growing patient group.

## 1. Introduction

Despite maximal treatment for glioblastoma (GB), outcomes remain poor with a median overall survival of 14.6 months with maximal surgical resection followed by the Stupp protocol of Temozolomide and radiotherapy [1]. GB has a median age of presentation of 64 years [2]. Advanced age is, however, not only a risk factor for GB, but also a negative prognostic indicator [3,4]. This poorer prognosis in older patients is likely multifactorial. Older patients are less likely to receive gross total resection (GTR), a key pillar for improved survival, as shown in a registry study of 20,705 patients [5]. Furthermore, significant medical comorbidities are more common in older patients and this is associated with poorer prognosis [6]. The presence of necrosis within the tumour is itself a negative prognosticator, and has also been shown to be age-dependent [7]. Finally, increasing age has been shown to independently predict poorer overall survival [8].

According to a UN report, the fastest-growing age demographic is those aged >65 years [9]. It follows that the incidence of GB should increase and this has indeed been observed [10]. The ageing population is associated with an increase in complex patients with advanced comorbidities, increasing frailty and dependence [11]. Despite this, the landmark Stupp trial did not include those aged >70 years [1]. In other areas of medicine, a difference in prognosis exists between those classified as youngest-old (~65–74 years), middle-old (~75–84 years), and oldest-old (>85 years) [12,13]. This distinction is likely of relevance in the neurosurgical management of GB in the older patients. The dilemma of how to manage older patients with GB will be faced more commonly by neurosurgeons. We therefore sought to evaluate the influence of age on survival in patients with gliolastoma, with a particular focus on patients aged ≥70 years.

## 2. Methods

We undertook a retrospective review of all histologically confirmed glioblastoma cases in a single tertiary neurosurgical centre over a period between 1st of January 2021 to 31st March 2025. All cases were IDH wild-type glioblastoma, diagnosed based on the 2021 WHO neuro-oncology classification as is routine practice in our institution. Only patients who had their index operation within this time window were included. Electronic medical records were reviewed to extract the following:

- Demographic variables (age at surgery, gender, smoking status);
- Smoking status was derived from clinical notes as any current or past smoking reported by the patient;
- Pre-operative Karnofsky Performance Status (KPS);
- Pre-operative Charlson Comorbidity Index (CCI);
- Surgical outcome: Gross total resection (GTR), subtotal resection (STR), biopsy;
- Tumour volume (cm^3^);
- Presence of new motor or speech deficit post-operatively;
- Methylation status (methylated/unmethylated);
- Adjuvant treatments received (Yes/No);
- Time to death or last follow-up (months) from surgery.

The extent of resection was defined based on the report of the post-operative MRI performed within 72 h of the operation. If there was any mention of residual contrast enhancing material that the reporting radiologist reported as possible tumour, then this was defined as subtotal resection. EOR was classified from contemporaneous post-operative MRI reports authored by specialist neuroradiologists and verified in the multidisciplinary team record.

The pre-operative tumour volume was calculating using the ABC/2 rule whereby the product of the maximum orthogonal antero-posterior, medio-lateral, and supero-inferior dimensions, measured on thin slice (0.5–1 mm) T1 + Contrast MR images were divided by two.

Adjuvant therapy was coded dichotomously (“Yes/No”) to indicate receipt of any post-operative radiotherapy and/or temozolomide, as detailed regimen information (fractionation, concurrent vs. sequential schedules, temozolomide-only) was inconsistently available. This approach reflects real-world documentation and possibly underestimates the true benefit of full-course chemoradiotherapy, since heterogeneous regimens were grouped together. Methylation status refers to MGMT promoter methylation determined by methylation-specific PCR using the institutional threshold for positivity. Results were classified as methylated, unmethylated, or unknown.

### Statistical Analysis

Descriptive statistics were used to summarise baseline demographic and clinical characteristics. Variables were reported as counts, mean with standard deviations, or medians with interquartile ranges (IQRs) and overall ranges where appropriate.

A cox proportional hazards regression model was used to explore the association between covariates and time-to-event, with time-to-event defined as the interval between surgery and either death or last follow-up. Only the index surgery was used for analysis given only a small number of cases with re-do operations. Continuous predictors were modelled on their natural scale. The survival function was visualised using Kaplan–Meier curves for the significant variables, and comparisons between groups were conducted using the log-rank test. Median survival times were derived for each subgroup. All Kaplan–Meier analyses were performed in R (version 4.4.2, Vienna, Austria) using the survival and survminer packages.

## 3. Results

**Figure 1:**
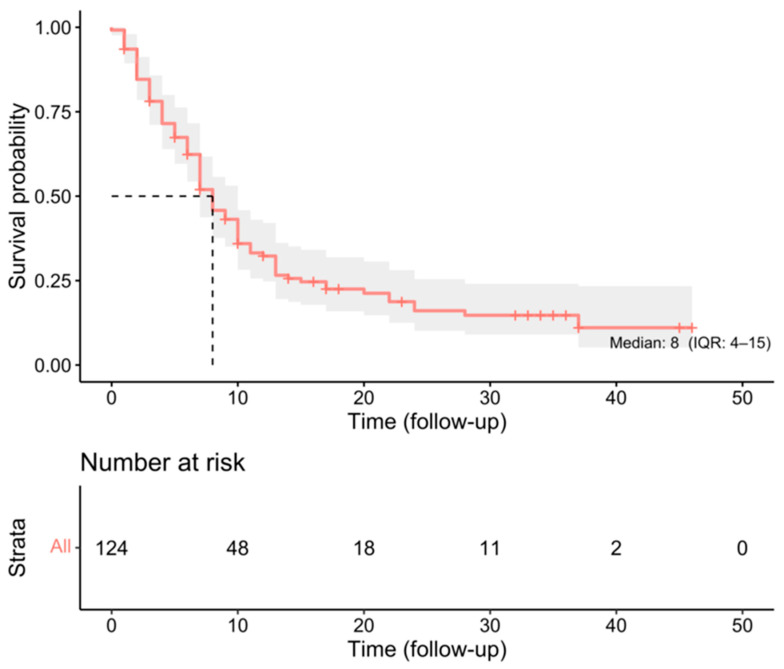
Kaplan–Meier survival curve for the whole cohort demonstrating a median survival of 8 months (dashed line).

124 patients were included in this study, with a median age of 74 years (range 70–86) with 53 (43%) females (Table 1). The median pre-operative KPS was 80 (range 20–90). The mean tumour volume was 28.9 cm^3^ (SD26.9 cm^3^). The median survival for the entire cohort was 8 months (IQR 4–15 months) (Figure 1). Nine patients in the cohort had re-do surgery within the inclusion time window.

### Factors Associated with Survival

**Figure 2:**
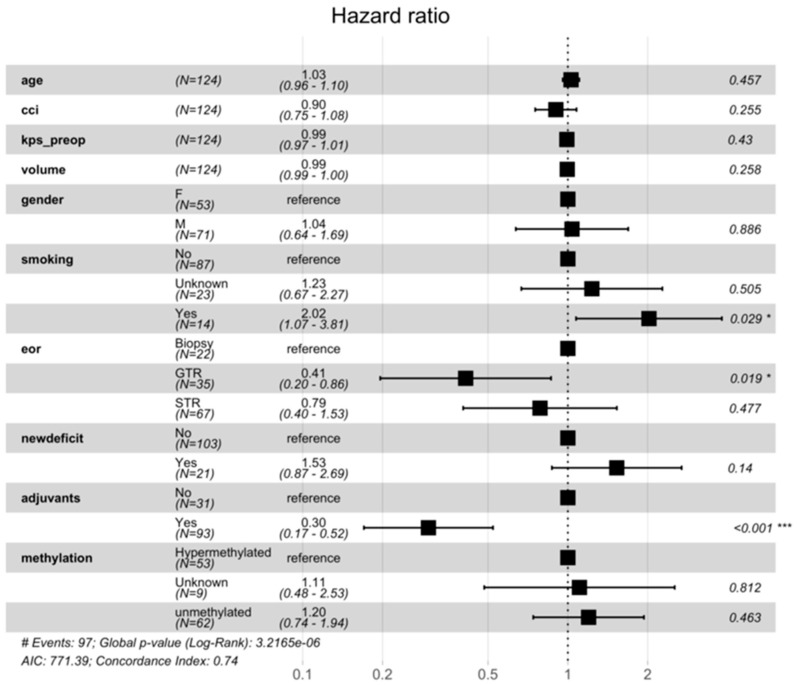
Forest plot indicating results of the cox proportional hazards model. In this multivariate assessment, significant predictors favouring better survival are GTR or STR and having received adjuvant chemoradiotherapy. Factors favouring worse survival include a history of smoking. Significance indicated using: * is *p* < 0.05, and *** is *p* < 0.001.

In the multivariable Cox proportional hazards model (Figure 2), adjuvant therapy was independently associated with improved overall survival (HR = 0.30, 95% CI 0.17–0.52; *p* < 0.001). Gross total resection (GTR), relative to biopsy, also reduced the hazard of death (HR = 0.41, 95% CI 0.20–0.86; *p* = 0.019), whereas subtotal resection (STR) showed no significant benefit (HR = 0.79, 95% CI 0.40–1.52; *p* = 0.48). Reparametrising the model to compare GTR with STR confirmed superior survival for GTR (HR = 0.52; *p* = 0.021).

To explore the absolute survival difference between GTR and STR, a restricted mean survival time (RMST) analysis was performed. The estimated mean survival advantage for GTR was 0.06 months at 6 months (95% CI −0.59–0.70; *p* = 0.86), 0.69 months at 12 months (95% CI −0.86–2.23; *p* = 0.38), and 2.01 months at 18 months (95% CI −0.48–4.50; *p* = 0.11). Although not statistically significant, the direction of effect favoured GTR at longer follow-up.

Smoking history was associated with increased mortality (HR = 2.02, 95% CI 1.07–3.81; *p* = 0.029). Sensitivity analyses addressing missing smoking data confirmed the robustness of this effect: HRs for smokers versus non-smokers remained around 1.9–2.0 with borderline significance (*p* = 0.036–0.052), and although attenuated when “unknowns” were treated as smokers (HR = 1.53; *p* = 0.089), the direction of association persisted.

No significant associations were observed for age, pre-operative KPS, Charlson Comorbidity Index, tumour volume, post-operative deficit, or methylation status (all *p* > 0.10).

Tests of the proportional hazards assumption showed non-proportionality for gender, smoking, extent of resection, and adjuvant therapy (global *p* = 0.0003). To address this, extended Cox models with time-by-covariate interactions were fitted. The effect of adjuvant therapy demonstrated a significant interaction with time (*p* = 0.0002), indicating that its survival benefit was greatest early post-operatively (HR ≈ 0.35 at 1 month) and diminished by 6–12 months (approaching HR ≈ 1). The effects of resection extent and smoking were time-stable (interaction *p* > 0.5). The time-averaged hazard ratio for adjuvant therapy remained strongly protective (HR = 0.34, 95% CI 0.19–0.63; *p* < 0.001).

**Figure 3:**
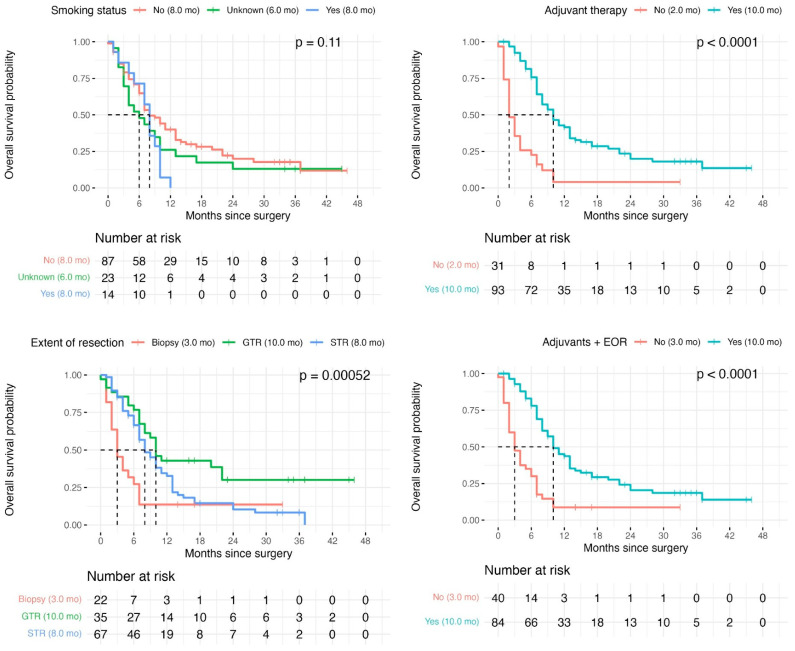
Kaplan–Meier curves to visualise the effect of the individually predictive variables. Median survival for each subgroup is in the brackets () within the figure legends. Top-left, effect of smoking history. Top right, effect of adjuvant therapies. Bottom-left, effect of extent of resection. Bottom-right, effect of being in the ‘ideal subgroup’ who received either GTR or STR plus adjuvant chemoradiotherapy. Dashed lines indicate median survival for each line/group.

On univariate analysis, median survival increased with resection extent—3 months for biopsy (95% CI 2–7), 8 months for STR (95% CI 8–NA), and 10 months for GTR (95% CI 7–11; *p* = 0.0005; Figure 3)—and with receipt of adjuvant therapy (10 months vs. 2 months; *p* < 0.001). Smoking was not significant on univariate comparison (*p* = 0.11). Patients receiving adjuvant therapy with either GTR or STR had the longest median survival (10 months, 95% CI 9–13) compared with 3 months (95% CI 2–6) in those who received neither.

**Figure 4:**
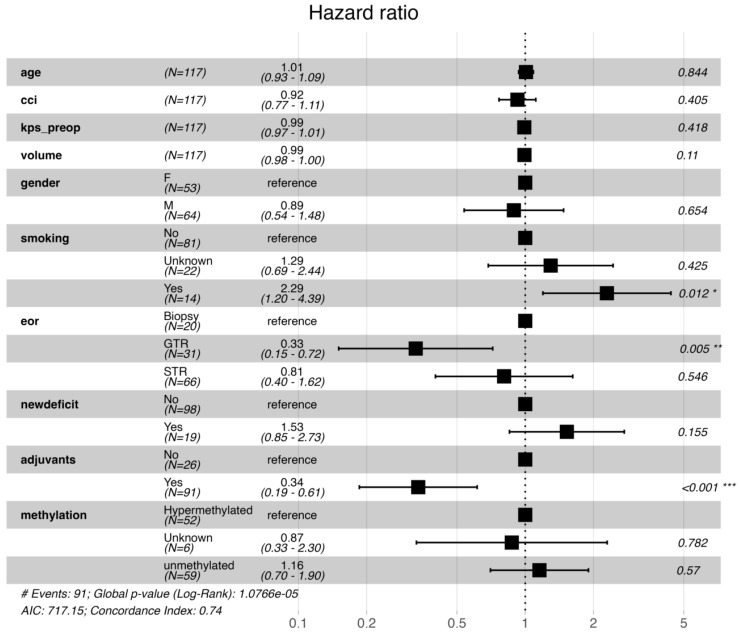
Sensitivity analysis excluding patients censored within 6 weeks post-surgery. The adjusted Cox model shows that adjuvant chemoradiotherapy and gross total resection remained independently associated with improved survival, while smoking history continued to predict poorer outcomes. Significance indicated using: * is *p* < 0.05, ** is *p* < 0.01, and *** is *p* < 0.001.

To account for potential selection bias (i.e., patients dying before receiving adjuvant therapy), analyses were repeated excluding individuals censored within 6 weeks post-surgery (Figure 4). Results were consistent: smoking remained detrimental (HR = 2.29; *p* = 0.012), while adjuvant therapy (HR = 0.34; *p* < 0.001) and GTR (HR = 0.33; *p* = 0.005) remained favourable predictors of survival.

## 4. Discussion

In this single-centre retrospective review of 124 patients aged >70 years with GB who received some form of surgical intervention, we identified an overall median survival of 8 months. Factors associated with survival were extent of resection, adjuvant chemoradiotherapy, and possibly smoking history. For subgroups receiving GTR or STR plus adjuvant chemoradiotherapy, survival modestly improved to a median of 10 months compared to 3 months for the biopsy-only group. Gross total resection was independently associated with a significantly lower hazard of death compared with subtotal resection; however, corresponding differences in restricted mean survival time were small and did not reach statistical significance, suggesting that any absolute survival benefit of GTR over STR is modest and uncertain in this cohort. The modest gain in survival with GTR/STR is important to relay in patient discussions. This is particularly relevant given that the additional time spent in hospital for surgery plus adjuvant treatments could take 2–3 months of this time, important factors for patients to consider in their decision-making.

Interestingly, pre-operative fitness (represented in the KPS and CCI) was not found to be a significant predictor or survival in either the multivariate or univariate assessment. However, in the cox proportional hazards model, a history of smoking appeared to be associated with worse survival, indicating the likely importance of lifestyle factors nonetheless.

It has long been recognised that overall, older patients with GB may have poorer prognosis than younger patients with median survival ranging from 4 to 9 months in other series [6,14,15,16]. The incidence of GB increases with age and with an ageing population across the developed world, neurosurgeons will face more older patients with GB. Despite this, the landmark Stupp trial for GB treatment was limited to patients aged 18–70 [1]. Similar restrictions excluding older patients was used for the Gliadel trial [17]. Nonetheless, randomised control trials focusing on older patients with GB have been undertaken such as the Perry et al. trial, which favourably evaluated the addition of temozolomide to short course radiation for GB in the older patients [18] and the Wick et al. trial evaluating temozolomide alone versus radiotherapy alone in older patients GB [19].

Patient selection in the older patients is made more challenging by the confounding effects of other variables with age that may influence survival such as comorbidities and poor performance status. In our series, we did not identify a significant statistical effect of KPS or CCI on survival. However, this is possibly owing to the already pre-selected nature of this cohort. This can be seen in the relatively low average CCI and high KPS in our study. This negative finding is also potentially owing to the fact that the CCI and KPS are not specific for this age group. A measure of physical and physiological reserve more suitable for this age group, such as a frailty score, may have better stratified patients. We also found that there was no significant difference in the survival of the GTR vs. the STR group. However, this may relate to our strict definition of GTR, that is GTR was recorded if absolutely no residual contrast enhancing material that could be a tumour was mentioned in the report. This definition may have led to more cases being labelled STR due to occasional mention of residual contrast enhancement that could be tumour or post-operative reactive change.

Several studies have evaluated the specific role of surgical resection in the older patients [3,4]. In other series, that extent of resection correlates with survival appears to remain the case with increasing age. A systematic review by Almenawer et al. [20] evaluating 12,607 older patients with high-grade glioma identified an improved overall survival of 14.4 months (95%CI 12.8–15.2) with gross total resection compared to 8.7 months (95%CI 7.9–9.5) with subtotal resection. Of note, the survival figure for the gross total resection group is similar to generally reported figures for GB survival. Using a prospectively collected database of 361 GB patients, Oszvald et al. showed a significantly reduced overall survival between patients aged <65 and >65 (median 14.9 vs. 9.1 months, respectively, *p* = 0.0001) [21]. However, when removing patients who underwent biopsy only, they found no difference in the survival of the two age groups (13.0 vs. 13.3 months, *p* = 0.86) indicating that age was not a prognostic factor in those patients who received gross total or subtotal resection. Halani et al. evaluated 120 patients with GB aged >65 years and showed that gross total resection was associated with an improved overall survival (14.1 vs. 9.6 months for subtotal resection, *p* = 0.038) [3]. This effect persisted when including all adjuvant therapies. However, Halani et al. identified 75 years as a predictive cut-off for poorer overall survival (7.9 vs. 15.1 months for those < 75 years, *p* < 0.0001). This age cut-off was significantly predictive even when controlling for extent of resection in multivariate modelling. Confirming this trend, Abdullah et al. found a poor overall survival of 4.2 months for those aged >80 excluding those receiving biopsy only [22].

The age cut-off above which is used to define ‘older patients’ varies and this confounds interpretation of the literature. Common definitions include >65 years as used by the National Institute for Health Research [23] and >60 as used by the World Health Organisation [24]. Mirroring this heterogeneity, in the above-mentioned systematic review by Almenawer et al., 35 studies covering 12,607 patients were included [20]. Across these studies, the definition of ‘older patients’ varied from >60 years (*n* = 8), >65 years (*n* = 15), >70 years (*n* = 9), and >75 years (*n* = 2) [20]. It is possible that varying definitions and low age cut-offs are masking cohorts with poorer prognosis. An important concept in this regard is the distinction between youngest-old (~65–74 years), middle-old (~75–84 years), and oldest-old (>85 years) patients. Unfortunately, given the limited number of patients aged >80 in our cohort, it is difficult for us to assess this distinction in our study. Several medical studies report variations in the prognoses between these age cut-offs for other medical conditions [13,25,26]. This distinction is likely of crucial importance in evaluating the prognosis in older patients GB. As identified in series such as those of Abdullah et al. [22] and Halani et al. [3], middle-old or oldest-old patients may have worse prognosis. We are likely to face increasing numbers of patients with GB in these age categories in the future. In our study, given the pre-selected nature of the cohort, the mean and median age was in fact quite low (74 years) relative to the range (up to age 86). This indicates a skewed dataset, likely reflecting natural patient selection. Further studies evaluating outcome specifically in the oldest-old cohorts are required to inform future practice.

### Limitations

This study is a single-centre retrospective series. The sample size was relatively small. Although we evaluated several confounding factors, residual confounding cannot be excluded, particularly with respect to comorbidities, frailty, and nuanced patient selection criteria that may have influenced both surgical decisions and outcomes. Molecular markers beyond methylation status were not analysed, which may have also contributed to prognostic heterogeneity. Owing to the pragmatic nature of the study, the pre-selected nature of the cohort meant the average age of the cohort was low, potentially missing effects of significantly advanced age on mortality. Finally, our study did not formally assess quality of life or functional outcomes, which are crucial considerations when counselling older patients regarding aggressive treatment strategies.

Also, EOR in this study was derived qualitatively from routine post-operative MRI reports rather than quantitative volumetric segmentation. Although this approach may misclassify small residuals and thus attenuate differences between GTR and STR, all scans were interpreted by specialist neuroradiologists within a single tertiary centre using standardised reporting conventions and multidisciplinary review. Any resulting bias would tend to diminish, rather than inflate, the apparent survival benefit of GTR. Accordingly, the observed association between GTR and improved hazard-adjusted survival, alongside only modest absolute RMST differences, likely represents a conservative estimate of the true effect size.

Adjuvant therapy was coded dichotomously (any post-operative radiotherapy and/or temozolomide) because specific regimen details were unavailable for the full cohort. This approach may underestimate the benefit of standard chemoradiotherapy, as less-intensive regimens were grouped together. Nevertheless, the strong overall association between receipt of adjuvant therapy and improved survival supports its prognostic significance even within this heterogeneous group. Methylation status refers to MGMT promoter methylation determined by methylation-specific PCR; incomplete reporting limited formal MGMT × treatment interaction analysis.

## 5. Conclusions

This study demonstrates that in patients aged ≥70 years with glioblastoma, overall survival remains limited with median survival of 8 months. The primary factors that influence survival are extent of resection and receipt of adjuvant chemoradiotherapy. While GTR or STR in combination with adjuvant treatments conferred a modest survival advantage, no clear benefit of more aggressive resection over STR was evident, underscoring the importance of tailored surgical strategies in this population. Although, pre-operative clinical fitness was not found to be a significant predictor, the potential association identified with smoking and worse prognosis indicates the need to consider lifestyle factors, a finding that may solidify in larger cohorts. Given the ageing population and rising incidence of glioblastoma in adults, prospective studies focusing on the “oldest-old” and incorporating both molecular variables and quality-of-life outcomes are desperately needed to better guide treatment decisions.

## Figures and Tables

**Table 1:** Summary of demographics of the cohort.

Variable	Mean (SD)	Median (IQR)	Range
Age	74.5 (3.4)	74 (72–77)	70–86
CCI	4.8 (1.5)	5 (4.0–5.2)	3–10
KPS pre-op	75.3 (11.7)	80 (70–80)	50–90
Volume	28.9 (26.9)	19.4 (9.9–43.9)	0.2–150.7
	Category	Count (percentage)	
Gender	Male	71 (57.3%)	
	Female	53 (42.7)	
Smoking history	Yes	14 (11.3%)	
	No	87 (70.2%)	
	Unknown	23 (18.5%)	
Extent of resection	GTR	35 (28.2%)	
	STR	67 (54.0%)	
	Biopsy	22 (17.7%)	
New post-op deficit	Yes	21 (16.9%)	
	No	103 (83.1%)	
Methylation	Hypermethylated	53 (42.7%)	
	Unmethylated	62 (50.0%)	
	Unknown	9 (7.3%)	
Adjuvant treatments	Yes	93 (75.0%)	
	No	31 (25.0%)	

CCI—Charlson Comorbidity Index. KPS—Karnofsky Performance Scale.

## Data Availability

Anonymised datasets will be made available upon reasonable request sent to the corresponding author.
